# Neuropsychiatric Disorders and Constipation: Unraveling Causal Links Through Genetic Analysis

**DOI:** 10.1002/brb3.71302

**Published:** 2026-03-05

**Authors:** Guojie Zhao, Haixia Ren, Yi Zhang, Zhi Wang, Qiao Yang, Shuang Liu, Minzhen Li, Zhiyu Xiang, Jingwen Liu

**Affiliations:** ^1^ Department of Neurosurgery, Chengdu Women's and Children's Central Hospital, School of Medicine University of Electronic Science and Technology of China Chengdu China; ^2^ Department of Gastroenterology Renmin Hospital of Wuhan University Wuhan Hubei Province China; ^3^ Sichuan Provincial Center For Mental Health, Sichuan Provincial People's Hospital, School of Medicine University of Electronic Science and Technology of China Chengdu China; ^4^ Key Laboratory of Psychosomatic Medicine Chinese Academy of Medical Sciences Chengdu China

**Keywords:** causal relationships, constipation, Mendelian randomization, neuropsychiatric disorders

## Abstract

**Background:**

Numerous observational studies have suggested a relationship between neuropsychiatric disorders and constipation. However, the specific causal relationships remain unclear. Mendelian randomization (MR) serves as a proven strategy for examining the causal relationships between genetic exposures and outcomes. In the present study, we used a two‐sample Mendelian randomization (TSMR) analysis to thoroughly explore the potential bidirectional genetic causal effects between neuropsychiatric disorders and constipation.

**Methods:**

We utilized the R11 data from Finnish genome‐wide association studies (GWAS) to examine the association between twelve common neuropsychiatric disorders and constipation using TSMR analysis. To establish this causal link, we applied the random‐effects inverse variance weighting (IVW) method. Additionally, we conducted various sensitivity analyses, including MR‐Egger analysis, weighted median analysis, and leave‐one‐out analysis, followed by heterogeneity testing. Finally, reverse MR testing was performed to further elucidate the potential causal relationship between constipation and neuropsychiatric disorders.

**Results:**

The forward MR results indicated that anxiety (IVW OR = 1.133; 95% CI: 1.021–1.258; *p* = 0.020) and depression (IVW OR = 1.149; 95% CI: 1.071–1.232; *p* = 0.000) may increase the risk of constipation. Reverse MR testing further confirmed that constipation increased the risk of anxiety (IVW OR = 1.273; 95% CI: 1.116–1.452; p = 0.000) and depression (IVW OR = 1.207; 95% CI: 1.095–1.331; *p* = 0.000) and was positively correlated with epilepsy (IVW OR = 1.331; 95% CI: 1.103–1.607; *p* = 0.003) and trigeminal neuralgia (IVW OR = 1.897; 95% CI: 1.225–2.936; *p* = 0.004). No pleiotropy or heterogeneity was observed in the MR analysis.

**Conclusion:**

Our research elucidates the underlying bidirectional causal relationship between twelve common neuropsychiatric disorders and constipation. These findings emphasize the importance for clinical practitioners to prioritize the identification and management of constipation symptoms in patients with neuropsychiatric conditions, aiming to enhance their overall health and quality of life.

## Graphical abstract

This study employed bidirectional two‐sample Mendelian randomization to explore the causal relationships between 12 neuropsychiatric disorders and constipation. Anxiety and depression might increase the risk of constipation, while constipation elevates the risks of anxiety, depression, epilepsy, and trigeminal neuralgia, emphasizing attention to constipation in neuropsychiatric care.

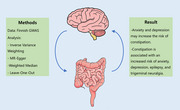

## 1 Introduction

Constipation is a prevalent clinical symptom significantly impacting patients' daily activities and overall quality of life (Rao et al. 2016). Insufficient dietary fiber intake, decreased physical activity, and cardiovascular diseases can increase the risk of constipation (Ogasawara et al. 2022). Individuals suffering from chronic constipation frequently experience repeated medical attention and may misuse laxatives, delaying treatment and increasing economic burden (Bharucha and Wald 2019). The prevalence of constipation is notably high among patients with neuropsychiatric disorders, as evidenced by multiple observational studies (Camilleri, 2021; Winge, 2003). Research has indicated that psychological factors, such as anxiety and depression, could affect gut function through efferent autonomic neural pathways (Emmanuel et al. 2001). Further, patients experiencing constipation are more likely to suffer from major depressive disorder (Wang et al. 2023). An NHANES‐based study identified mild depression as a significant predictor of constipation (Ballou et al. 2019). In addition, constipation has been reported in severe psychiatric disorders such as schizophrenia and bipolar affective disorder (Gong et al. 2024; Tazaki et al. 2024). Clinical research suggests that constipation is one of the most common gastrointestinal symptoms of Parkinson's disease, with over 50% of patients experiencing constipation during their illness (Yao et al. 2023). Meta‐analyses and cohort studies have highlighted a close association between constipation and the risk of developing Parkinson's disease (Adams‐Carr et al. 2016; Svensson et al. 2016). A bi‐national cohort study has indicated that constipation increases the risk of Alzheimer's disease, with slow intestinal transit potentially being a key mechanism in Alzheimer's disease pathogenesis (Kang et al. 2023). Moreover, studies show a positive correlation between the rates of constipation and the prevalence of dementia (Zhang et al. 2018). In stroke rehabilitation wards, 60% of patients suffer from constipation (Harari et al. 2004). Patients with multiple sclerosis frequently suffer from gastrointestinal dysfunction, alternating between constipation and fecal incontinence (Bourre et al. 2023; Preziosi et al. 2018). Constipation is also a common comorbidity in epilepsy patients and may act as a trigger for seizures (Gabrielsson et al. 2023; Beck et al. 2021). However, observational studies and cross‐sectional surveys may be influenced by confounding factors, such as reverse causation, which could hinder accurate assessments of the relationship between neuropsychiatric disorders and constipation.

Mendelian randomization (MR) is a commonly used method in epidemiological research to strengthen causal inference. It utilizes genetic variations as instrumental variables (IV) to assess the causal relationship between exposure and outcome. This method effectively mitigates confounding factors and reverse causation that are inherent in observational studies, while also addressing representativeness and feasibility issues that may arise in RCTs (Skrivankova et al. 2021). Although recent studies have shown an association between psychosomatic disorders such as anxiety and depression and constipation (Murray et al. 2020; Wu et al. 2024), no study has systematically employed MR analysis to investigate the causality between a range of common neuropsychiatric disorders and constipation.

In this research, we investigated potential causal relationships between twelve neuropsychiatric disorders and constipation, including anxiety, depression, bipolar affective disorder, Parkinson's disease, Alzheimer's disease, and trigeminal neuralgia. We employed bidirectional TSMR using the Finnish database to establish a theoretical foundation for the treatment and management of patients with neuropsychiatric disorders.

## 2 Materials and Methods

### 2.1 Research Design

This study employed data from a genome‐wide association study (GWAS) to explore the potential causal relationship between 12 neuropsychiatric disorders and constipation using two‐sample Mendelian randomization analysis, supplemented by sensitivity analyses to assess the robustness of the findings. Mendelian randomization research necessitates adherence to three core assumptions: (a) the instrumental variable must be strongly associated with the exposure; (b) the instrumental variable should not be related to any confounding factors that may affect the exposure‐outcome relationship; and (c) the instrumental variable can only influence the outcome variable through the exposure factor. Given that publicly available data were utilized, ethical review and patient informed consent were not required.

### 2.2 Data Sources

All genome‐wide association study (GWAS) data for our phenotypes were sourced from the Finnish database. To minimize the risk of population stratification bias, the data were exclusively obtained from individuals of European descent, as illustrated in **Table** **1**.

**TABLE 1:** Brief information about the GWAS database research data in this study.

Phenotype	ID	Number of case	Number of controls	Number of SNPs	Population
Anxiety disorders	finngen_R11_F5_ALLANXIOUS	3178	40,3817	21,306,523	European
Depression	finngen_R11_F5_DEPRESSIO	53,313	394,756	21,306,683	European
Bipolar affective disorders	finngen_R11_F5_BIPO	8209	394,756	21,305,816	European
Schizophrenia	finngen_R11_F5_SCHZPHR	6933	439,144	21,306,637	European
Parkinson's disease	finngen_R11_G6_PARKINSON	5150	448,583	21,306,794	European
Alzheimer disease	finngen_R11_G6_ALZHEIMER	11,755	441,978	21,306,794	European
Stroke	finngen_R11_C_STROKE	47,841	3280,79	21,305,386	European
Multiple Sclerosis	finngen_R11_G6_MS	2620	449,770	21,306,740	European
Hydrocephalus	finngen_R11_G6_HYDROCEPH	2642	420,369	21,306,300	European
Epilepsy	finngen_R11_G6_EPLEPSY	14,089	341,884	21,304,789	European
Migraine	finngen_R11_G6_MIGRAINE	23,899	341,884	21,305,028	European
Trigeminal neuralgia	finngen_R11_G6_TRINEU	1973	395,832	21,305,028	European
Constipation	finngen_R11_K11_CONSTIPATION	44,590	409,143	21,306,794	European

### 2.3 Selection Criteria for Genetic Variants

To meet the three core assumptions necessary for Mendelian randomization studies, we first selected single nucleotide polymorphisms (SNPs) significantly associated with the exposure factor at a genome‐wide level (*p* < 5 × 10^−^^8^, *r* ^2^ < 0.001, genetic distance = 10,000 KB). Subsequently, we excluded SNPs exhibiting significant heterogeneity based on heterogeneity testing, ultimately retaining those SNPs that were significantly associated with the exposure factor as instrumental variables. To evaluate whether the selected instrumental variables were subject to weak instrument bias, we calculated the F‐statistic, excluding SNPs with an *F*‐statistic of less than 10.

### 2.4 Data Analysis

This study primarily employed random‐effects inverse variance weighting (IVW) along with supplementary methods, including weighted median, MR‐Egger, weighted mode, and simple mode, for sensitivity analysis to evaluate the validity and robustness of the IVW results. Odds ratios (OR) were used to assess the potential causal relationships between twelve neuropsychiatric disorders and constipation, with a significance threshold set at *p* < 0.05. To avoid statistical significance driven by trivial effects, this study incorporates the Region of Practical Equivalence (ROPE) to distinguish between statistical significance and practical relevance. The ROPE range is defined as log OR ∈ [−0.18, 0.18] (equivalent to OR: 0.83 − 1.19), an empirical threshold widely adopted in epidemiology, medical statistics, and Bayesian analysis (Schönbrodt and Wagenmakers 2018; Zou et al. 2024). We used the leave‐one‐out method for sensitivity analysis to determine the robustness of the results. To mitigate bias from horizontal pleiotropy, we applied the MR‐Egger intercept test to detect pleiotropy within the data, with a *p*‐value < 0.05 indicating the presence of pleiotropy. Finally, to further assess the robustness of statistically significant findings, we utilized Cochran's *Q* test *p*‐value to evaluate heterogeneity in the analysis, with a Cochran *Q*‐derived *p* ≥ 0.05 suggesting no heterogeneity in the causal analysis.

## 3 Results

### 3.1 Causal Effect of Neuropsychiatric Disorders on Constipation

The Mendelian randomization results for 12 neuropsychiatric disorders as exposures and constipation as an outcome are presented in **Table** **2**. We observed that anxiety (IVW OR = 1.133; 95% CI: 1.021–1.258; *p* = 0.020) and depression (IVW OR = 1.149; 95% CI: 1.071–1.232; *p* = 0.000) were significantly associated with an increased risk of constipation. Furthermore, results from supplementary MR methods including weighted median, MR‐Egger, weighted mode, and simple mode were consistent with the IVW estimates, all indicating positive causal effects. Specifically, eight single nucleotide polymorphisms (SNPs) were selected as instrumental variables for anxiety and 25 SNPs for depression.

**TABLE 2:** Mendelian randomization analysis results (12 neuropsychiatric disorders as exposures, constipation as the outcome).

Exposure	Outcome	N(SNPs)	Method	OR(95%CI)	*p*‐value
Anxiety disorders	Constipation	8	IVW	1.133 (1.021–1.258)	0.020
			MR Egger	1.148 (0.783–1.682)	0.507
			Weighted median	1.153 (1.013–1.312)	0.031
			Simple mode	1.178 (0.978–1.420)	0.128
			Weighted mode	1.179 (0.975–1.427)	0.134
Depression		25	IVW	1.149 (1.071–1.232)	0.000
			MR Egger	1.514 (1.116–2.056)	0.014
			Weighted median	1.143 (1.038–1.258)	0.006
			Simple mode	1.202 (1.004–1.439)	0.057
			Weighted mode	1.094 (0.918–1.306)	0.323
Bipolar affective disorders		20	IVW	1.053 (1.003–1.106)	0.038
			MR Egger	1.034 (0.900–1.189)	0.641
			Weighted median	1.064 (1.007–1.125)	0.026
			Simple mode	1.065 (0.969–1.170)	0.205
			Weighted mode	1.063 (0.965–1.173)	0.230
Schizophrenia		12	IVW	1.020 (0.987–1.054)	0.243
			MR Egger	1.052 (0.983–1.127)	0.175
			Weighted median	1.014 (0.970–1.061)	0.532
			Simple mode	1.011 (0.937–1.091)	0.784
			Weighted mode	1.011 (0.931–1.098)	0.801
Parkinson's disease		4	IVW	1.027 (0.932–1.132)	0.590
			MR Egger	1.040 (0.760–1.423)	0.829
			Weighted median	1.025 (0.955–1.100)	0.491
			Simple mode	0.952 (0.803–1.127)	0.606
			Weighted mode	0.950 (0.821–1.100)	0.545
Alzheimer disease		17	IVW	1.019 (0.963–1.077)	0.010
			MR Egger	1.019 (0.987–1.053)	0.271
			Weighted median	1.036 (1.009–1.063)	0.009
			Simple mode	1.019 (0.963–1.077)	0.526
			Weighted mode	1.034 (1.011–1.058)	0.010
Stroke		13	IVW	1.082 (0.992–1.179)	0.075
			MR Egger	0.864 (0.684–1.090)	0.243
			Weighted median	0.996 (0.893–1.111)	0.944
			Simple mode	0.972 (0.777–1.216)	0.808
			Weighted mode	0.976 (0.862–1.105)	0.705
Multiple sclerosis		11	IVW	1.001 (0.978–1.024)	0.942
			MR Egger	1.014 (0.973–1.057)	0.519
			Weighted median	1.015 (0.992–1.039)	0.215
			Simple mode	0.962 (0.905–1.023)	0.248
			Weighted mode	1.020 (0.996–1.044)	0.128
Hydrocephalus		4	IVW	0.995 (0.920–1.077)	0.907
			MR Egger	1.729 (0.875–3.418)	0.256
			Weighted median	0.996 (0.942–1.052)	0.875
			Simple mode	0.943 (0.825–1.078)	0.453
			Weighted mode	0.949 (0.834–1.079)	0.481
Epilepsy		21	IVW	1.046 (0.987–1.108)	0.131
			MR Egger	1.013 (0.896–1.146)	0.835
			Weighted median	1.053 (0.975–1.137)	0.188
			Simple mode	1.091 (0.927–1.283)	0.306
			Weighted mode	1.082 (0.915–1.279)	0.368
Migraine		9	IVW	1.045 (0.948–1.152)	0.374
			MR Egger	1.078 (0.685–1.695)	0.756
			Weighted median	1.073 (0.959–1.202)	0.218
			Simple mode	1.152 (0.938–1.414)	0.215
			Weighted mode	1.117 (0.942–1.325)	0.239
Trigeminal neuralgia		5	IVW	0.966 (0.925–1.008)	0.112
			MR Egger	1.040 (0.932–1.160)	0.537
			Weighted median	0.939 (0.886–0.995)	0.032
			Simple mode	0.934 (0.856–1.019)	0.198
			Weighted mode	0.933 (0.849–1.026)	0.227

### 3.2 Causal Effect of Constipation on Neuropsychiatric Disorders

When we conducted a reverse Mendelian randomization analysis with constipation as the exposure factor and twelve neuropsychiatric disorders as the outcomes, the results were shown in Table 3. We identified 19 SNPs as instrumental variables and observed that constipation may be associated with the occurrence of anxiety (IVW OR = 1.273; 95% CI: 1.116–1.452; *p* = 0.000), depression (IVW OR = 1.207; 95% CI: 1.095–1.331; *p* = 0.000), epilepsy (IVW OR = 1.331; 95% CI: 1.103–1.607; *p* = 0.003), and trigeminal neuralgia (IVW OR = 1.897; 95% CI: 1.225–2.936; *p* = 0.004). Furthermore, supplementary methods, including weighted median, MR‐Egger, weighted mode, and simple mode, were consistent with the direction of the IVW, all indicating a positive effect.

**TABLE 3:** Mendelian randomization analysis results (constipation as the exposure, 12 neuropsychiatric disorders as the outcomes).

Exposure	Outcome	N(SNPs)	Method	OR(95%CI)	*p*‐value
Constipation	Anxiety disorders	19	IVW	1.273 (1.116–1.452)	0.000
			MR Egger	1.167 (0.766–1.778)	0.481
			Weighted median	1.191 (1.002–1.416)	0.047
			Simple mode	1.338 (0.958–1.867)	0.104
			Weighted mode	1.226 (0.891–1.687)	0.227
	Depression	19	IVW	1.207 (1.095–1.331)	0.00
			MR Egger	1.395 (1.029–1.892)	0.046
			Weighted median	1.141 (1.003–1.296)	0.044
			Simple mode	1.089 (0.855–1.387)	0.460
			Weighted mode	1.089 (0.871–1.361)	0.465
	Bipolar affective disorders	19	IVW	1.199 (0.959–1.499)	0.111
			MR Egger	0.980 (0.487–1.977)	0.958
			Weighted median	1.211 (0.900–1.630)	0.207
			Simple mode	1.296 (0.717–2.341)	0.402
			Weighted mode	1.271 (0.741–2.181)	0.394
	Schizophrenia	19	IVW	1.021 (0.721–1.446)	0.905
			MR Egger	1.657 (0.554–4.963)	0.378
			Weighted median	0.970 (0.611–1.540)	0.897
			Simple mode	0.853 (0.349–2.084)	0.732
			Weighted mode	0.827 (0.360–1.901)	0.660
	Parkinson's disease	19	IVW	1.279 (0.981–1.668)	0.067
			MR Egger	0.734 (0.319–1.686)	0.476
			Weighted median	1.182 (0.812–1.720)	0.382
			Simple mode	1.209 (0.627–2.333)	0.578
			Weighted mode	1.194 (0.607–2.346)	0.614
	Alzheimer disease	19	IVW	1.222 (0.939–1.589)	0.136
			MR Egger	1.829 (0.800–4.180)	0.170
			Weighted median	1.233 (0.923–1.646)	0.156
			Simple mode	1.280 (0.671–2.440)	0.463
			Weighted mode	1.261 (0.697–2.282)	0.454
	Stroke	19	IVW	1.072 (0.956–1.202)	0.234
			MR Egger	0.955 (0.663–1.376)	0.809
			Weighted median	1.078 (0.931–1.248)	0.316
			Simple mode	1.118 (0.847–1.475)	0.442
			Weighted mode	1.125 (0.851–1.487)	0.418
	Multiple sclerosis	19	IVW	1.135(0.786–1.639)	0.499
			MR Egger	1.880 (0.597–5.920)	0.295
			Weighted median	1.107 (0.660–1.856)	0.701
			Simple mode	1.109 (0.485–2.537)	0.809
			Weighted mode	1.109 (0.473–2.602)	0.815
	Hydrocephalus	19	IVW	0.138 (0.915–1.907)	0.138
			MR Egger	1.970 (0.622–6.240)	0.265
			Weighted median	1.388 (0.819–2.360)	0.226
			Simple mode	1.697 (0.641–4.490)	0.301
			Weighted mode	1.662 (0.610–4.532)	0.333
	Epilepsy	19	IVW	1.331 (1.103–1.607)	0.003
			MR Egger	2.215 (1.284–3.823)	0.011
			Weighted median	1.423 (1.121–1.806)	0.004
			Simple mode	1.441 (0.896–2.317)	0.149
			Weighted mode	1.415 (0.889–2.252)	0.161
	Migraine	19	IVW	1.143 (0.981–1.331)	0.087
			MR Egger	0.975 (0.599–1.587)	0.921
			Weighted median	1.209 (0.997–1.465)	0.053
			Simple mode	1.241 (0.858–1.794)	0.267
			Weighted mode	1.235 (0.872–1.748)	0.249
	Trigeminal neuralgia	19	IVW	1.897 (1.225–2.936)	0.004
			MR Egger	2.634 (0.650–10.678)	0.193
			Weighted median	2.440 (1.372–4.339)	0.002
			Simple mode	2.677 (1.026–6.985)	0.060
			Weighted mode	2.677 (1.032–6.945)	0.058

### 3.3 Sensitivity Analysis

**FIGURE 1:**
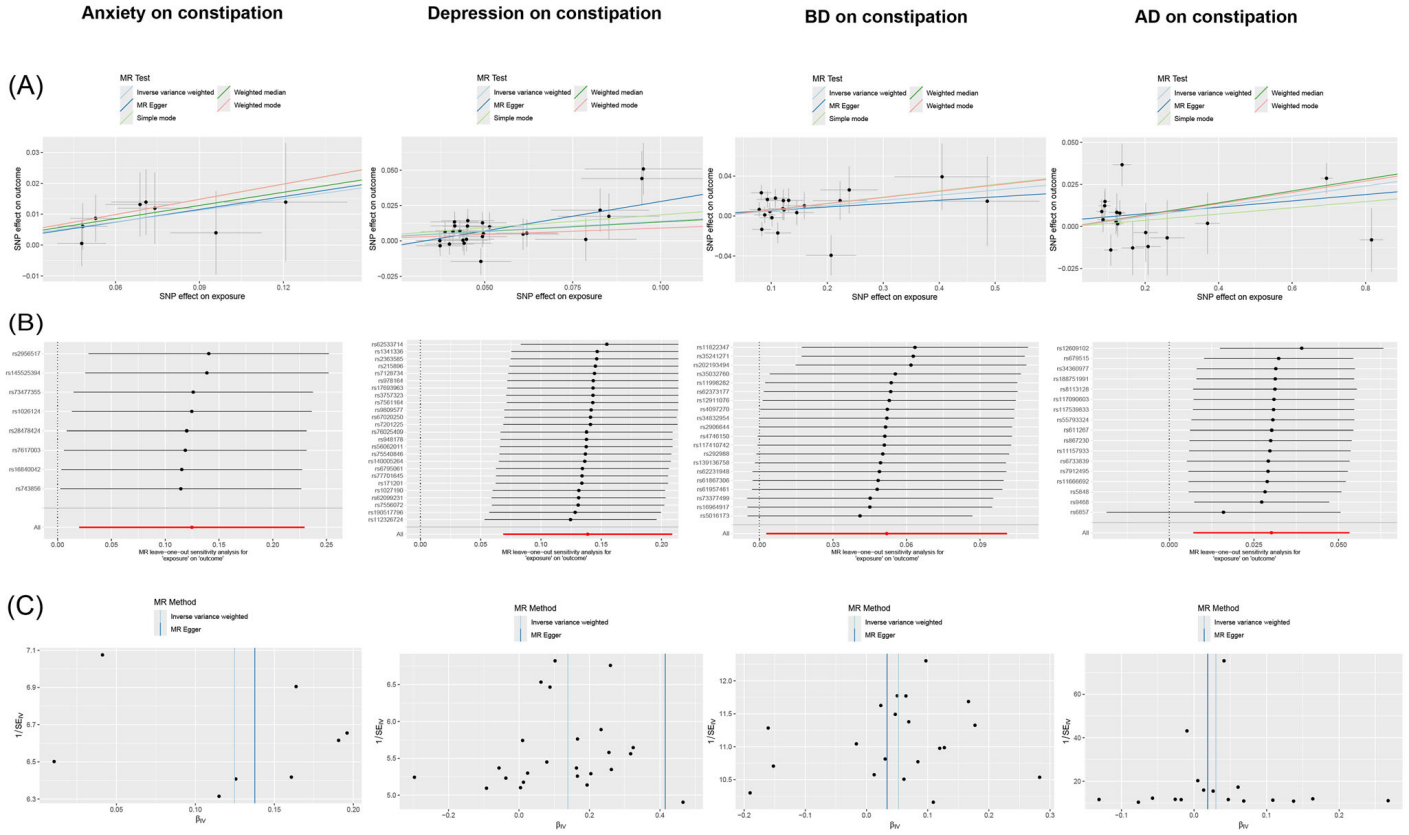
Twelve neuropsychiatric disorders as exposures and constipation as the outcome. (A) The scatter plots of MR analyses, (B) The leave‐one‐out plots for assessing the robustness, and (C) The funnel plots for assessing heterogeneity.

**FIGURE 2:**
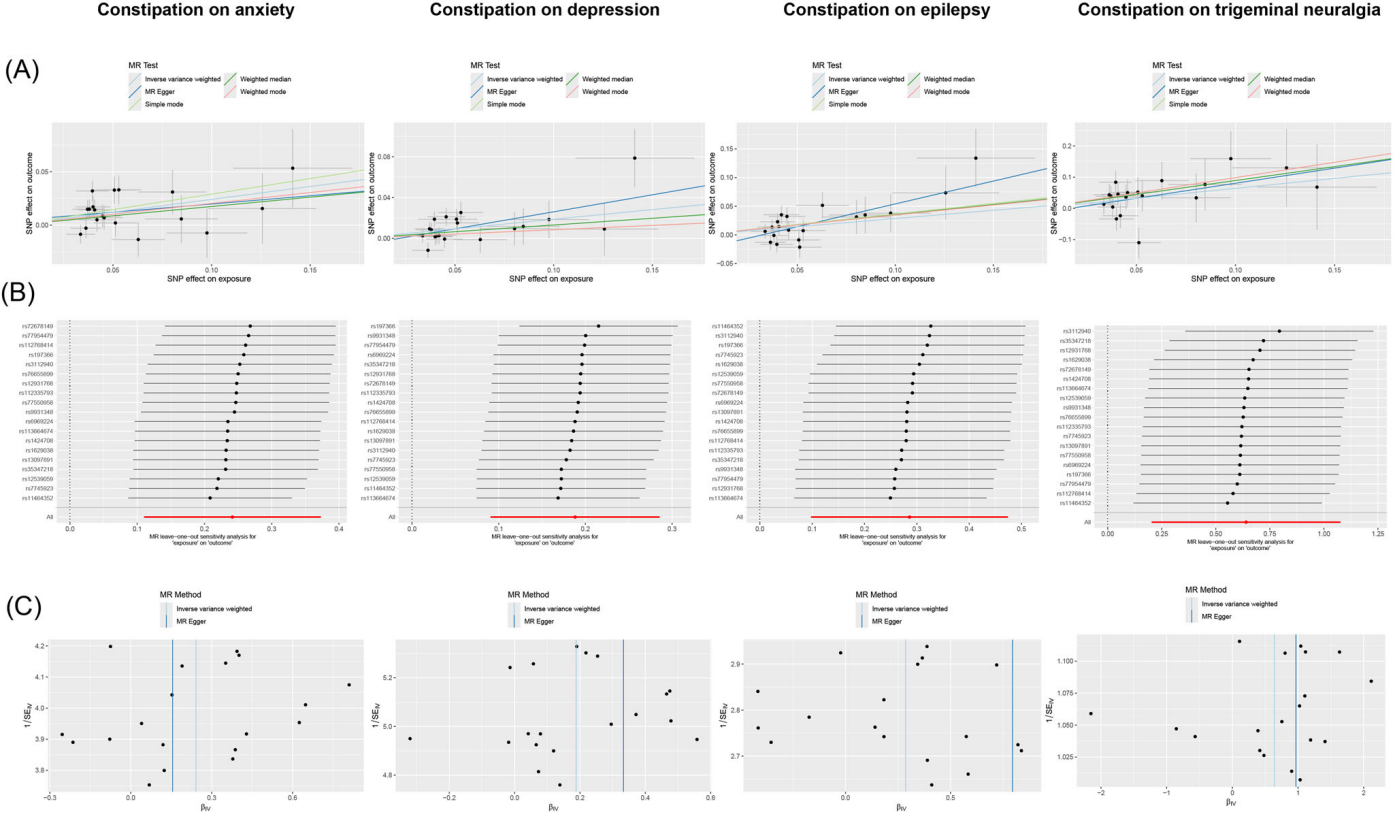
Constipation as the exposure and 12 neuropsychiatric disorders as the outcomes. (A) The scatter plots of MR analyses, (B) The leave‐one‐out plots for assessing the robustness, and (C) The funnel plots for assessing heterogeneity.

We further conducted horizontal pleiotropy and heterogeneity tests for the phenotypes with positive results, as summarized in Tables 4 and 5. As shown in the tables, we found no evidence of horizontal pleiotropy or heterogeneity. We visualized the results of all analyses, creating scatter plots, leave‐one‐out plots, and funnel plots in Figures 1 and 2.

**TABLE 4:** Horizontal pleiotropy (MR‐Egger intercept test).

Exposure	Outcome	Egger‐Intercept	*p*‐value
Constipation	Anxiety disorders	0.004	0.676
	Depression	−0.007	0.338
	Epilepsy	−0.025	0.070
	Trigeminal neuralgia	−0.016	0.634
Anxiety disorders	Constipation	−0.001	0.948
Depression		−0.014	0.082
Bipolar affective disorders		0.002	0.787
Alzheimer disease		0.004	0.343

**TABLE 5:** Heterogeneity tests (Cochran's *Q* test results).

Exposure	Outcome	Method	*Q*‐value	*p*‐value
Constipation	Anxiety disorders	IVW	24.393	0.142
		MR Egger	24.136	0.116
	Depression	IVW	21.584	0.251
		MR Egger	20.418	0.253
	Epilepsy	IVW	24.529	0.138
		MR Egger	20.110	0.269
	Trigeminal neuralgia	IVW	19.164	0.381
		MR Egger	18.902	0.334
Anxiety disorders	Constipation	IVW	1.447	0.984
		MR Egger	1.442	0.963
Depression		IVW	21.859	0.588
		MR Egger	18.546	0.727
Bipolar affective disorders		IVW	29.466	0.059
		MR Egger	29.343	0.044
Alzheimer disease		IVW	22.564	0.126
		MR Egger	21.208	0.130

## 4 Discussion

The data indicate that genetic susceptibility to anxiety disorder and depression may increase the risk of constipation. Based on the analysis using the ternary decision rule, regarding the association between anxiety disorder and constipation, the 95% ​CIs of the OR values obtained by all analytical methods overlap with the region of ROPE. This suggests that the current evidence is insufficient to confirm the clinically significant causal effect of anxiety disorders on constipation. However, due to the limitation of statistical power, the possibility of a weak association between the two cannot be completely ruled out.​ In the analysis of the association between depression and constipation, the results obtained by different methods show discrepancies; the OR values from the IVW method and the weighted median method fall within the ROPE, while the OR value from the MR‐Egger method lies outside the ROPE. This result suggests that depression may exert a weak enhancing effect on the risk of constipation through mechanisms independent of the emotional pathway. The contradictory nature of the aforementioned results indicates that the conclusions of the main analysis may be disturbed by pleiotropic factors, and their reliability needs to be further verified through additional research. Results from the reverse study indicated that the genetic susceptibility to constipation was associated with the onset of anxiety, depression, epilepsy, and trigeminal neuralgia. However, we found no genetic‐level correlation between neuropsychiatric disorders (e.g., Parkinson's disease, multiple sclerosis) and constipation, despite prior observational evidence supporting this association.

Constipation is a common clinical symptom characterized by irregular or difficult bowel movements. This condition significantly affects patients' quality of life and increases healthcare burdens (Stern and Davis 2016; Cifci et al. 2023). Neuropsychiatric disorders are prevalent brain diseases with complex etiologies. Both genetic and non‐genetic factors contribute to disease susceptibility, imposing a significant economic burden on patients' families, healthcare systems, and nations (Kennedy et al. 2003). Our Mendelian randomization study based on the Finnish R11 database suggests that there may be a bidirectional causal relationship between constipation and anxiety disorder as well as depression, which is consistent with the findings of previously published observational studies (Cheng et al., 2003). A meta‐analysis indicated that the risk of Alzheimer's disease increased among patients with constipation, potentially due to alterations in the gut environment triggering neurodegenerative diseases through the gut‐brain axis, although our findings did not support a clinically meaningful causal relationship between Alzheimer's disease and constipation (Fu et al. 2020). Furthermore, we identified an association between constipation and an increased risk of epilepsy and trigeminal neuralgia. Our study found no causal relationship between stroke and constipation, contradicting some cross‐sectional studies but aligning with prior Mendelian randomization findings regarding the relationship between constipation and stroke (Sun et al. 2022; Camara‐Lemarroy et al. 2014). Similarly, regarding the relationship between Parkinson's disease and constipation, the results do not support a causal association, conflicting with earlier observational studies (Svensson et al. 2016). Several factors may account for the differences between our findings and previously published research. First, observational studies can suggest associations between diseases but may not establish causality. Constipation may share common underlying factors with stroke and Parkinson's disease, such as lifestyle and age, while our Mendelian randomization study effectively controlled for these confounding variables at the genetic level (Boden‐Albala et al. 2011; Fu et al. 2022). Second, our study population may differ from that of the observational study in terms of gender and age structure. Such differences may influence or obscure the relationship between constipation and stroke or Parkinson's disease, leading to different results (Burgess et al. 2012).

Numerous studies have reported gastrointestinal motility disorders among patients with neuropsychiatric disorders, with constipation being the most common (Xu et al. 2021). Several physiological mechanisms may clarify the causal relationship between neuropsychiatric disorders and constipation. Research indicated that mood disorders could affect intestinal motility, consequently leading to constipation (Gorard et al. 1996). Conversely, constipation may stimulate the release of pro‐inflammatory cytokines, compromising the integrity of the intestinal barrier and triggering systemic chronic low‐grade inflammation (Gao et al. 2022). Additionally, pro‐inflammatory cytokines activate the serotonin reuptake transporter (SERT) in the gut, leading to reduced serotonin levels, potentially explaining constipation's role in the development of depression (Mittal et al. 1996; Shatri et al. 2023). The gut microbiota plays a crucial role in both human physiology and pathology. Recent studies have shown that various neuropsychiatric disorders exhibit alterations in gut microbiota, including depression, anxiety, autism, schizophrenia, Parkinson's disease, Alzheimer's disease, stroke, epilepsy, and multiple sclerosis (Sorboni et al. 2022; Zhuang et al. 2018). Research suggested that gut microbiota imbalance may contribute to constipation, mainly characterized by a decline in microbial diversity and abundance, a reduction in beneficial bacteria, and an increase in harmful bacteria (Fu et al. 2021; Dimidi et al. 2017). The causal relationship between constipation and neuropsychiatric disorders may be mediated by gut microbiota dysbiosis. Moreover, a bidirectional communication pathway between the gut microbiota and the brain, known as the gut‐brain axis, has been proposed (Guevara‐Gonzaléz et al. 2022). Several studies have confirmed that dysregulation of gut‐brain axis signaling is closely associated with the onset of various neurodegenerative and psychiatric disorders (Anand et al. 2022). The complex microbial ecosystem of the gastrointestinal tract regulates central nervous system function through microbial actions and the gut‐brain axis. In addition, research has suggested that probiotics are a promising treatment strategy for various neuropsychiatric disorders and constipation (Liu et al. 2019; Dimidi et al. 2017; Naomi et al. 2021). The gut‐immune‐brain axis has emerged as a new field of research into the mechanisms of neuropsychiatric diseases, potentially revealing how constipation impacts these disorders (Delaney and Hornig 2018).

This study represents the first systematic exploration of the causal relationship between neuropsychiatric disorders and constipation. We employed a bidirectional two‐sample MR method, providing robust causal inference for our findings. Due to ethical constraints, implementing this experimental design was not feasible within randomized controlled trials (RCTs). Furthermore, achieving a sufficient number of endpoint events in RCTs often necessitates large sample sizes and long‐term follow‐up, which may not always be practical. Second, our two‐sample MR approach was based on existing large‐scale GWAS data, ensuring our findings were not confounded by behavioral, social, psychological, or other factors. While previous Mendelian randomization studies have identified causal links between constipation and certain psychiatric disorders, the present study possesses distinct advantages. First, most prior investigations focused solely on associations between constipation and a single or small number of neuropsychiatric disorders (e.g., exclusively examining the depression‐constipation relationship). In contrast, our study incorporates twelve common neuropsychiatric disorders (including anxiety, depression, bipolar affective disorder, Alzheimer's disease, epilepsy, and trigeminal neuralgia), thereby comprehensively covering high‐incidence and high‐impact disease entities. Second, using a bidirectional two‐sample MR design, we not only clarified the positive causal effects of specific neuropsychiatric disorders on constipation but also confirmed the reverse causal impacts of constipation on some neuropsychiatric disorders, thus revealing their complex bidirectional causal relationships. Third, we utilized GWAS datasets distinct from those in previous research. Consistency verification across different databases strengthens the conclusions of earlier studies and enhances the robustness of the causal associations identified. Therefore, our research outcomes hold significant clinical value and offer new perspectives for disease management. However, this study has several limitations. First, the study population consists solely of individuals of European descent, limiting the generalizability of our findings. Second, various neuropsychiatric disorders and constipation exhibit different clinical subtypes, and this study did not perform subgroup analyses to determine specific causal relationships among the subtypes. Future research should conduct more reliable large‐sample, multi‐center clinical studies to elucidate these causal relationships further.

## 5 Conclusion

Our research elucidates the underlying bidirectional causal relationship between twelve common neuropsychiatric disorders and constipation. These findings emphasize the importance for clinical practitioners to prioritize the identification and management of constipation symptoms in patients with neuropsychiatric conditions, aiming to enhance their overall health and quality of life.

## Author Contributions

**Guojie Zhao**: conceptualization, data curation, formal analysis, methodology, resources, software, and writing – original draft. **Haixia Ren**: funding acquisition, methodology, resources, and writing – original draft. **Yi Zhang**: investigation, resources, and visualization. **Zhi Wang and Qiao Yang**: visualization. **Shuang Liu, Minzhen Li, and Zhiyu Xiang**: resources. **Jingwen Liu**: formal analysis, funding acquisition, project administration, supervision, validation, writing – original draft, writing – review and editing.

## Funding

This study was supported from the National Nature and Science Youth Fund of China (No. 82200598).

## Ethics Statement

The data analyzed in this study are de‐identified data obtained from GWAS summary data, and the ethics approval and consent to participate are not required.

## Conflicts of Interest

The authors declare no conflicts of interest.

## Data Availability

The data that support the findings of this study are available from the corresponding author upon reasonable request.
